# Barrett's esophagus stages: their correlation with SBS17-associated DNA mutations and the identification of histological marker genes

**DOI:** 10.1080/23723556.2022.2026559

**Published:** 2022-01-22

**Authors:** Georg A. Busslinger

**Affiliations:** a Division of Gastroenterology and Hepatology, Department of Internal Medicine III, Medical University of Vienna, Vienna, Austria; b Research Center for Molecular Medicine (CeMM) of the Austrian Academy of Sciences, Vienna, Austria

**Keywords:** Barrett's esophagus, single-cell RNA and DNA analyses, single-base substitution 17 (SBS17A and SBS17B)

## Abstract

We have recently reported a correlation between the accumulation of specific T > C and T > G mutations and the chromosomal instability in cells of Barrett's esophagus (BE), which represents a premalignant condition of esophageal adenocarcinoma. Additionally, we identified seven marker genes that facilitate the distinction of individual BE stages by histopathological examination.

## Author's comment

The incidence of Barrett's esophagus (BE) increased in the last decades and affects up to 11% of all adults. Chronic gastroesophageal reflux is considered a major risk factor, which leads to the replacement of the healthy multi-layered esophageal epithelium with a single-layered columnar epithelium in the distal esophagus. BE is detected during routine endoscopy, and its diagnosis is verified by histology. BE is a premalignant condition of esophageal adenocarcinoma (EAC) and is classified into different stages.^1^ It is, however, still debated whether the presence of intestinal metaplasia (IM), as measured by the appearance of goblet cells, is a prerequisite for the diagnosis of BE. Patients with IM are believed to have a higher risk of EAC development. The different BE stages are columnar epithelium without IM, non-dysplastic BE (NDBE), low-grade dysplasia (LGD) and high-grade dysplasia (HGD). No curative treatment for BE exists, and the only option is to endoscopically resect more advanced BE stages such as LGD and HGD. The distinction of the individual stages relies on histopathological assessment of morphological anomalies and remains challenging.

Different approaches were applied to identify stage-specific marker genes for histological assessment, although with little success. Single-cell RNA-sequencing (scRNAseq) is an attractive approach to identify such marker genes. While others focused their analyses on NDBE biopsies^2^ or did not specify the respective BE stages,^3^ we performed scRNAseq experiments with biopsies corresponding to different BE stages.^4^ Our analysis identified two sets of marker genes that distinguish between BE with and without IM, and *CLDN2* as a marker gene for dysplastic BE stages. Next to scRNAseq, we used, to our knowledge for the first time, single-cell DNA-sequencing (scDNAseq) to detect chromosomal instabilities (CINs) and DNA mutations at single-cell resolution in BE. Interestingly, only cells with CINs accumulated specific T > C and T > G mutations known as the COSMIC mutational signature SBS17. This finding adds to our current knowledge of BE pathology by identifying an early mutational event, which likely contributes to later tumor development.

Initially, we established organoid cultures from different anatomic locations of the same patient, including the non-diseased esophageal epithelium, gastric cardia, corpus, pylorus and LGD BE. These cultures were clonally expanded for whole-genome sequencing (WGS), which revealed SBS17-associated mutations only in BE organoids but not in organoids from the non-diseased control tissues. The mechanism causing the SBS17-specific mutations is still poorly understood. Notably, these alterations are primarily detected in gastric and esophageal cancers.^5^ We wondered if all cells in the BE tissue are equally affected by the underlying mutagenic mechanism. We therefore performed scDNAseq experiments with fourteen biopsies from eight patients.^4^ Our high-quality scDNAseq data facilitated the determination of megabase-wide chromosomal rearrangements and the identification of genome-wide mutational patterns, while the detection of gene-specific mutations was impossible. All NDBE biopsies were chromosomally stable (CS) and were void of SBS17 signatures. One of the two LGD biopsies and all analyzed HGD samples revealed multiple cell clusters, which carried different degrees of CINs, while one cluster was always CS. The observed clonal heterogeneity is consistent with previously published WGS experiments.^6^ Interestingly, DNA mutations corresponding to SBS17 were only observed in the CIN clusters in five of six biopsies but never in CS cell clusters. This difference was most obvious, when analyzing NDBE and HGD biopsies from the same patients, as SBS17-specific mutations were detected only in CIN cells of the HGD biopsy but not in NDBE cells. The identification of this relationship was possible due to the single-cell resolution of the scDNAseq method^4^ and could not be observed by WGS analyses.^6^,^7^ As the mutagenic process causing the SBS17-specific mutations is still unknown, it is tempting to speculate that chronic gastroesophageal reflux may be involved, although it cannot explain the selective mutagenesis observed in only a subset of cells. Either the accumulation of SBS17-specific mutations is uncoupled from the gastroesophageal reflux or it acts together in concert with a second, still unknown mechanism.

To identify stage-specific marker genes, we performed single-cell RNA sequencing (scRNAseq) experiments with replica plates corresponding to the scDNAseq experiments and additionally with new biopsies. We analyzed eighteen biopsies from fourteen patients and compared them to our previously published scRNAseq data of healthy control epithelium.^8^ BE cells revealed the best transcriptional overlap with gastric epithelium as also shown by others.^3^ Unexpectedly, BE cells did not form clusters according to their BE stages.^4^ Some clusters were even biopsy-specific, which may reflect differences in the underlying CIN patterns. Although we attempted to correct for these CIN biases, we failed in identifying novel BE stage-specific marker genes. A plausible explanation is that the expression of genes in chromosomal stable regions is indirectly influenced by the deregulation of genes located in CIN regions. Since our dataset was composed of all the different BE stages, we selected seventeen genes from different clusters and analyzed their expression by RNA *in situ* hybridization experiments on histological sections from endoscopic resections containing distinct BE stages within the same slide. Seven genes proved suitable to distinguish between BE stages. Selective expression was observed for *PSCA, SLC5A5* and *LIPF* in columnar epithelium without IM, for *ANPEP, CEACAM6* and *REG4* in BE with IM and for *CLDN2* in dysplastic BE stages. Notably, our RNA *in situ* hybridization method is more sensitive and specific than previous antibody staining, which yielded controversial results.^9^,^10^ Our gene expression data will need to be confirmed in a larger patient cohort to corroborate the utility of these new marker genes for histopathological assessment.
